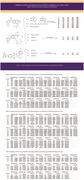# Inhibitory profile and selectivity of novel ROCK 2 inhibitors: an in‐silico study

**DOI:** 10.1002/alz70859_103089

**Published:** 2025-12-25

**Authors:** Kimia Mohammadi

**Affiliations:** ^1^ Kimiagar Toos Pharmaceutical Company, Mashhad, Khorasan Razavi Iran (Islamic Republic of)

## Abstract

**Background:**

Protein phosphorylation, regulated by protein kinases, is essential for numerous cellular processes. Rho‐associated kinases 1 and 2 (ROCKs), key serine/threonine kinases and downstream effectors of RhoA, play significant roles in cell growth and apoptosis. ROCK signaling is implicated in various diseases, including neurodegenerative disorders, where its activation exacerbates disease progression. Inhibition of ROCK 2 has shown potential therapeutic benefits, such as enhancing tau autophagy, improving dendritic spine density, promoting Aβ clearance, and reducing neuroinflammation. This study focuses on developing ROCK 2‐selective inhibitors to aid Alzheimer’s patients. However, the structural similarity between ROCK 1 and ROCK 2 makes selectivity challenging. To address this, we used virtual screening to assess inhibitor selectivity across 23 kinases, aiming to minimize side effects.

**Method:**

We designed 63 compounds using ChemDraw, inspired by three known and research ROCK2‐selective inhibitors (SAR407899 (S homologs), N‐(2‐(2‐(dimethylamino)ethoxy)‐4‐(1H‐pyrazol‐4‐yl)phenyl)acetamide (P homologs), and, N‐(4‐(1H‐pyrazol‐4‐yl)phenyl)chromane‐3‐carboxamide (C homologs)) and and functional groups known to influence ROCK inhibition. Protein X‐ray structures of 23 kinases—including ROCK1, ROCK2, MEK1, MEK2, JNK1, P38 isoforms (α, β, γ, δ), MAPKAPK2, MSK1, PKA, PKB, PKC isoforms (α, θ), PDK1, SGK1, GSK3β, AMPK, CK2, PhK, LCK, and ChK1—were retrieved from the Protein Data Bank (PDB). The designed compounds were docked into the active sites of these proteins. Top‐performing inhibitors were identified based on docking scores (>9 for S and P homologs and >8 for C homologs), and their inhibitory constants (Ki) were analyzed at room temperature. A compound was considered selective if its Ki ratio was greater than 10.

**Result:**

The analysis of the top 17 compounds showed varying levels of selectivity toward ROCK 2 compared to other kinases. The most selective compound, Peic, demonstrated a Ki > 10 for 19 proteins, indicating high selectivity toward these targets. while Si emerged as the least selective overall, displaying ki>10 only for 4 proteins.

**Conclusion:**

Out of the 63 compounds, the top 17 showed promising potency and selectivity for 4 to 19 kinases. The strong performance and selective nature of these compounds make them potential candidates for treating Alzheimer’s disease by reducing its symptoms with fewer side effects.